# Tunable Release of Curcumin with an In Silico-Supported Approach from Mixtures of Highly Porous PLGA Microparticles

**DOI:** 10.3390/ma13081807

**Published:** 2020-04-11

**Authors:** Concetta Di Natale, Valentina Onesto, Elena Lagreca, Raffaele Vecchione, Paolo Antonio Netti

**Affiliations:** 1 Center for Advanced Biomaterials for Health Care (CABHC), IstitutoItaliano di Tecnologia, Largo Barsanti Matteucci 53, 80125 Napoli, Italy; Concetta.dinatale@iit.it (C.D.N.); Valentina.onesto@iit.it (V.O.); Elena.lagreca@unina.it (E.L.); paoloantonio.netti@unina.it (P.A.N.); 2 Interdisciplinary Research Centre on Biomaterials (CRIB), University of Naples Federico II, P.leTecchio 80, 80125 Naples, Italy; 3 Department of Chemical Materials and Industrial Production (DICMAPI), University of Naples Federico II, P.leTecchio 80, 80125 Naples, Italy

**Keywords:** PLGAMPs, drug delivery, curcumin, in silico, release model, first-order equation

## Abstract

In recent years, drug delivery systems have become some of the main topics within the biomedical field. In this scenario, polymeric microparticles (MPs) are often used as carriers to improve drug stability and drug pharmacokinetics in agreement with this kind of treatment. To avoid a mere and time-consuming empirical approach for the optimization of the pharmacokinetics of an MP-based formulation, here, we propose a simple predictive in silico-supported approach. As an example, in this study, we report the ability to predict and tune the release of curcumin (CUR), used as a model drug, from a designed combination of different poly(d,l-lactide-co-glycolide) (PLGA) MPs kinds. In detail, all CUR–PLGA MPs were synthesized by double emulsion technique and their chemical–physical properties were characterized by Mastersizer and scanning electron microscopy (SEM). Moreover, for all the MPs, CUR encapsulation efficiency and kinetic release were investigated through the UV–vis spectroscopy. This approach, based on the combination of in silico and experimental methods, could be a promising platform in several biomedical applications such as vaccinations, cancer-treatment, diabetes therapy and so on.

## 1. Introduction

In recent decades, polymeric microparticles (MPs) have been widely used as drug delivery systems for the controlled release of small molecules, proteins or peptides [1,2,3,4,5]. The reason of this great diffusion is due to several attractive features such as the use of non-laborious techniques [6], low production costs [7], simplicity in industrial scale-up [8] and possibilities of different ways of administration (oral, ocular, parental, inhalation) [9,10]. Particularly, biodegradable MPs composed of PLGA, a random copolymer of poly(glycolic acid) (PGA) and poly(lactic acid) (PLA), are well-established drug delivery systems for small macromolecules involved in the treatment of several important diseases including cancer [11]. PLGA is also a Food and Drug Administration (FDA) and European Medicine Agency (EMA) approved polymer for ophthalmologic, and other medical applications. PLGA shows relatively high miscibility with other polymers and solvents [11], and, moreover, it is able to encapsulate both hydrophilic and lipophilic drugs [12]. The use of PLGA MPs has many advantages, including biocompatibility, non-immunogenicity, non-toxicity, and the possibility of monitoring the polymer degradation in physiological environments to obtain a controlled drug delivery [12]. Particularly, PLGA biodegradation involves a hydrolytic and an auto-catalytic degradation which includes hydration, hydrolytic degradation, auto-catalytic degradation and solubilization [11,13,14]. Usually, the degradation time decreases by increasing the glycoside units content, but the pharmacokinetics characteristics of PLGA MPs are defined also by other factors such as initial MW [7,14], the monomer composition ratio of the PLGA matrix [15,16], stereochemistry (composition in D and L), end-group functionalization [17], drug type [18] and pH value of the release medium [1,15,17,19,20]. In addition, the porosity of MPs can influence the drug release; for example, a relatively high porosity facilitates water penetration into particles leading to a direct drug release from the porous and faster MPs degradation resulting again in a faster drug release [21]. By contrast, a low degree of porosity can hinder water diffusion into MPs reducing the rate of polymer degradation [22].

The combination of these complex factors, without understanding their kinetics and dynamics, makes it difficult to predict and control the release of a drug. 

Mathematical models represent a fundamental tool to optimally design new pharmaceutical systems, to study drug formulations and to evaluate in vitro and in vivo releases [23,24,25,26]. They rely on the model fitting of experimental data and equations and they enable a quantitative interpretation of the values obtained from a drug release assay [27]. Release systems can be divided into those that release drug following a slow zero-or first-order kinetics and in controlled release patterns that provide an initial burst, followed by a slow zero-or first-order release of the sustained drug, to maintain as long as possible the desired pharmaceutical concentration in the target tissues or in the blood [28]. In this regard, the shape and dimension of the system designed to achieve a specific drug release profile as well as the amount and type of the active agent, adjuvants and polymer can be predicted by mathematical models, in order to obtain a fine control on the drug release kinetics [27]. 

In silico models can help to understand more deeply the physical and chemical mechanisms of drug release reducing also the number of experiments. They are particularly useful to analyze different conditions and strategies when the effects of diverse phenomena are joined [29]. For example, mathematical modeling and parametric analysis were used to analyze the temperature effects of the controlled drug release process from PLGA biodegradable nanoparticles [30]. 

Usually, empirical or mechanistic models are applied in the drug delivery field and in particular, the second ones, taking into account both physical and chemical phenomena that occur during the drug release, show a very high predictive ability towards specific experimental conditions [31,32].

In this study, we aim to develop a smart approach, based on the combination of in silico and experimental methods, to predict and control the release of a specific drug. In particular, we chose curcumin as a model drug and encapsulated it into PLGA MPs in three different ways: as water phase, as oil phase and as nano-emulsion. We decided to use curcumin since it is a powerful active substance by itself (ex. anti-inflammatory [33,34], anticancer [33]) and a hydrophobic molecule with a logP value of ~3.0, which allows it to be dissolvable in common organic solvents and partially soluble in polar solvents including water [35]. These characteristics are common to many hydrophobic drugs (prostaglandin, doxorubicin) making curcumin an ideal model drug. Additionally, these features allowed us to perform three different kinds of encapsulation, providing different kinds of release, which then could be combined to obtain tunable kinetic release profiles in a predictive way. 

The MPs were synthesized by double emulsion technique and their chemical–physical properties were characterized by Mastersizer, UV–vis and microscopy technologies. In the end, a non-linear first-order in silico model was used to predict and tune (mixing the proper quantity of the three formulations) its release from MPs which was later confirmed by the experimental data. We think that the use of an in silico model, in combination with experimental data, could be very useful to design MPs with desired drug release profiles in order to control therapeutic dosages. 

## 2. Materials and Methods 

### 2.1. Materials

Poly (lactic-co-glycolic acid) (PLGA) RESOMER® RG 504 H, 38,000–54,000 Dalton,lactide–glycolide = 50:50, was purchased from Boehringer Ingelheim (Ingelheim am Rhein, Kreis, Germany) Curcumin (*Curcuma longa*, Turmeric, powder, M.W. = 368.38), dichloromethane (DCM), ammonium bicarbonate (ABC), dimethyl sulfoxide (DMSO), Mowiol® 40–88 (poly(vinyl)alcohol (PVA) MW 27,000–32,000 PVA) and Tween 20 were purchased from Sigma-Aldrich (Milan, Italy). Soybean oil and Lipoid E80 lecithin were purchased from Lipoid (Lipoid AG, Steinhausen/ZG, Switzerland) Bidistilled water was pretreated with a Milli-Q R Plus System (Millipore Corporation, Bedford, OH, USA). 

### 2.2. Method 

#### 2.2.1. CUR–MPs Production 

##### CUR in Oil Phase (CUR–Oil) and Water Phase (CUR–Water) Formulation

CUR–MPs were prepared by the water/oil/water double emulsion/solvent evaporation technique as already reported [36]. In particular, a gas foaming porous agent, ammonium bicarbonate (ABC), at a concentration of 7.5 mg/mL was added in the first emulsion to obtain highly porous particles. Twelve milligrams of curcumin were loaded in DCM for the oil phase preparation, while the same amount of drug was dissolved in 100 µL of ethanol and 900 µL of water plus 1 mL of DMSO in the water phase formulation.

##### CUR-o/w 20% Oil Nano-Emulsion (CUR–NE) as Water Phase 

The CUR–NE was prepared as previously reported [33,37]. Briefly, 1.2 mg of egg-lecithin (surfactant) was dissolved in 5 mL of soybean oil (oil phase). After, 20.83 mg of CUR was added to the mixture [38]. The final emulsion was obtained by adding 19.3 mL of Milli-Q water to oil phase [34]. After the process, 100 µL of CUR–NE was used to produce CUR–NE-MPs as described in the previous paragraph.

#### 2.2.2. CUR–MPs Characterization 

##### Confocal Microscopy

All three CUR–MPs formulations were characterized by confocal microscopy (Leica SP5 microscope (Wetzlar, Germania)) in order to evaluate the signal of the molecule inside them. In detail, fluorescence images were acquired using an HCX IRAPO L 40×/0.95 water objective and a 488 nm laser as an excitation source as already described [39].

##### Microparticle Size and Polydispersity Index (PDI)

The mean size and the PDI of all CUR–MPs were determined by static light scattering (Mastersizer 3000, Malvern Instruments, Malvern, UK) using a concentration of 3 mg/mL in water.

##### Scanning Electron Microscopy (SEM)

CUR–MPs morphology was evaluated by SEM microscopy as already described [40]. Concisely, 20 µL were deposited on a standard SEM pin stub and analyzed by FESEM ULTRA-PLUS (Zeiss) (Milan, Italy) at 5 kV with the SE2 detector. Moreover, the internal porous structure of the MPs was investigated using a PDMS 2 mm in thickness cured at 80 °C for 30 min. After cooling, MPs were deposited on it and another PDMS layer 2mm in thickness was used to cover them up. Finally, the solid PDMS block was frozen in liquid nitrogen (–196 °C) and sectioned using the Leica CryoUltra Microtome EM-FC7-UC7 (Milan, Italy). 

#### 2.2.3. Entrapment Efficiency (%ɳ) of CUR inside MPs 

The %ɳ of curcumin inside the three formulations of MPs was measured dissolving 10 mg of MPs in 1 mL of DMSO for 30 min, at room temperature. The solution was then analyzed by UV–vis (UV–Visible-V-730 UV–Visible Spectrophotometer, Jasco, (Cremella, (LC), Italy) following the signal at 426 nm. The quantity of curcumin-loaded was obtained through the Beer–Lambert law using 58,547 dm^3^·mol^−1^·cm^−1^ as the molar extinction coefficient of curcumin in DMSO [41]. All experiments were performed in triplicate.

#### 2.2.4. In Vitro Release Study 

##### In Silico Approach

Curcumin in oil phase, water phase and nano-emulsion experimental release data were fitted using MATLAB®(v.R2019a) (Turin, Italy) employing an exponential growth model. In particular, the curcumin release $C_{r}$ was described by:

(1) $$C_{r}=a\left(1-e^{-bt}\right)$$

where a and b are the model parameters, and y=0 at t=0 the initial conditions.

A simple release kinetics prediction under the non-linear first-order assumption of Equation (1) was developed by Equation (2):

(2) $$\begin{array}{c} C_{r}=\frac{\sum_{1}^{n}C_{n}a_{n}\left(1-e^{-b_{n}t}\right)}{\sum_{1}^{n}C_{n}}\\ \mathrm{with}\overset{n}{\underset{1}{\sum }}C_{n}=C_{1}+C_{2}+\ldots +C_{n}=100 \end{array}$$

where $a_{n}$ and $b_{n}$ are the model parameters, $C_{n}$ is the percentage of weighted curcumin MPs, n is the number of different MP formulations considered. 

##### In Vitro Cumulative Release of CUR from MPs

In vitro curcumin release profile was obtained by the UV–vis technique (V-730 UV–Visible Spectrophotometer, Jasco, Cremella, (LC), Italy)).Aliquots of 5 mg of the three different microparticle formulations were suspended in 1.5 mL of phosphate buffer saline PBS at pH 7.2, vortexed under magnetic stirring at 550 rpm and incubated at 37 °C. At defined time intervals, 1 mL of PBS was removed without removing particles. The supernatants were then diluted 1:1 in ethanol and analyzed by UV–vis using as molar extinction coefficient 28,648 dm^3^·mol^−1^·cm^−1^ [42].The experiments were achieved in triplicate.

## 3. Results and Discussion

### 3.1. CUR—Microparticle Production and Morphological Characterization

**Figure 1:**
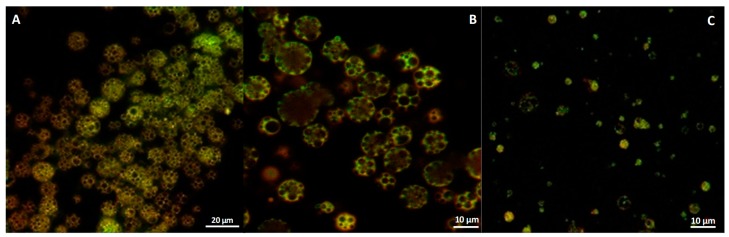
Confocal images of curcumin-loaded microparticles: (**A**) curcumin (CUR)–oil, (**B**) CUR–water and (**C**) CUR–nano-emulsion (NE).

CUR–MPs were produced through the double emulsion technique using the method of solvent evaporation [36]. Three different configurations were obtained (CUR–NE, CUR–oil and CUR–water) in order to produce microspheres with curcumin molecules embedded inside or outside the porous structures. This strategy was designed to produce MPs with different drug release kinetics in physiological conditions. To compare the differences between the microparticles, their morphology was evaluated using Confocal and SEM microscopies. In particular, as shown in Figure 1A,B, CUR–oil and CUR–water microparticles were visibly homogeneous with a high fluorescent signal corresponding to the embedded curcumin molecules. In detail, in the oil configuration, all curcumin was outside the porous structure as expected, while in the water conformation most of it was located inside the pores. Some aggregation phenomena were instead visible in the CUR–NE microparticles, probably due to the instability of the nano-emulsion during the production process (Figure 1C). 

**Figure 2:**
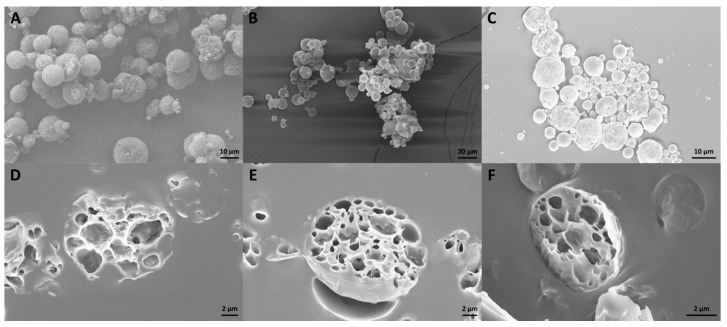
SME microscopy of microparticles (MPs) microstructure. (**A**) CUR–oil, (**B**) CUR–water and (**C**) CUR–NE. In addition, their internal porosity was evaluated, depositing them on a PDMS layer 2 mm in thickness and cutting a PDMS block: (**D**–**F**) CUR–oil, CUR–water and CUR–NE, respectively.

Similar considerations can be made analyzing microparticles by SEM microscopy. In general, CUR–oil and CUR–water microparticles showed a homogeneous polymeric surface (Figure 2A,B) and by investigating their internal porous structure it was possible to confirm an open porosity for both the strategies of production (Figure 2D,E). As for CUR–NE microparticles, they displayed good open porosity as the configurations just described (Figure 2F) but their polymeric surface showed a slightly porous structure (Figure 2C), maybe some nano-emulsion droplets aggregated on the surface generating a closed superficial porosity.

**Figure 3:**
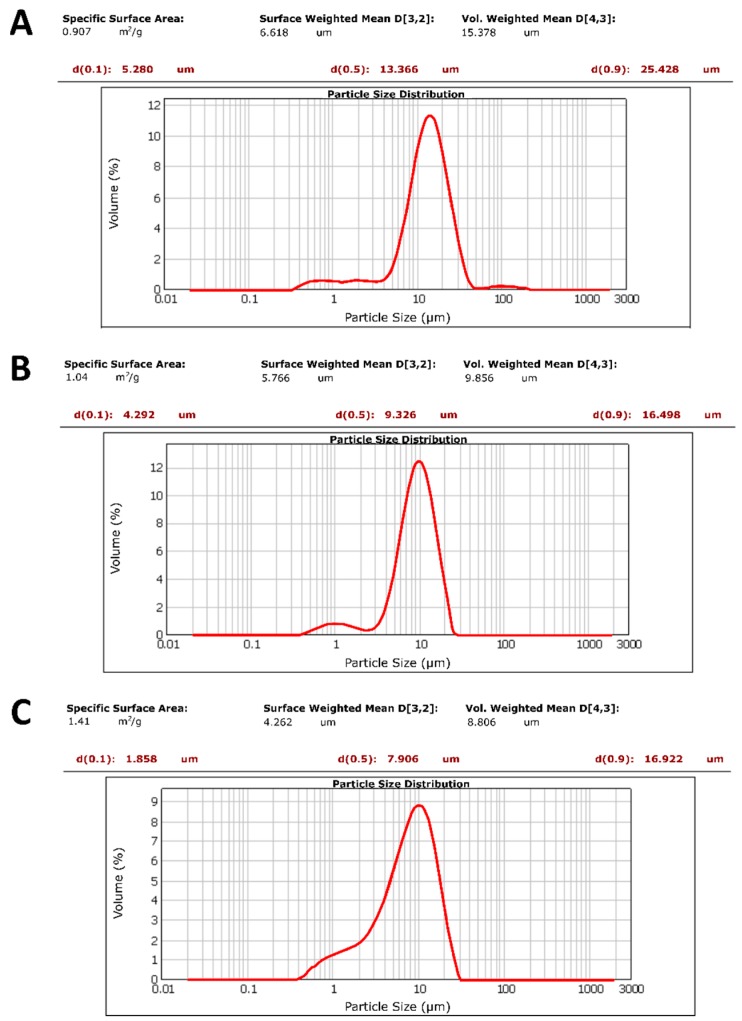
CUR–MPs dimensional analysis by Mastersizer at 3 mg/mL in water solution: (**A**) CUR–oil, (**B**) CUR–water and (**C**) CUR–NE microparticles.

The homogeneity of MPs was also confirmed by analyzing their size with a Malvern Mastersizer. The obtained results showed that CUR–oil and CUR–water microparticles have a uniform distribution with a mean diameter of 13.36 µm and 9.32 µm (Figure 3A,B).Contrarily, as for the CUR–NE, despite having an average diameter of 7.9 µm, they expose a less sharp curve with a large peak at ≅1 µm typical of the nano-emulsion used in this study, corroborating our hypothesis about its instability during the production phase of the microparticles (Figure 3C).

### 3.2. %ɳ of CUR inside MPs

In order to evaluate the amount of curcumin encapsulated into MPs, 10 mg of each formulation was dissolved in a basic solution as reported in the Materials and Methods section. Table 1summarizes the %ɳ for all three preparations; in particular, CUR–oil and CUR–water reached encapsulation efficiencies of 40.01 ± 0.3 and 42.30 ± 3.5, respectively. As for the CUR–NE, the %ɳ was 31.02 ± 0.5 and it was maybe due to the instability of the nano-emulsion during the microparticle production steps (e.g., high speed, pH, time for solvent evaporation) as argued in the previous paragraph.

### 3.3. Release Study

#### 3.3.1. In Silico Prediction

Release rate studies are important to control, tune and adjust the drug dose during a time-long therapy such as for diabetes [43], chemotherapy [44] and other chronic diseases as neurological [45] or inflammation diseases [46,47].To this end, mathematical modeling can provide valuable information on the mechanism of the release process [25]. 

**Figure 4:**
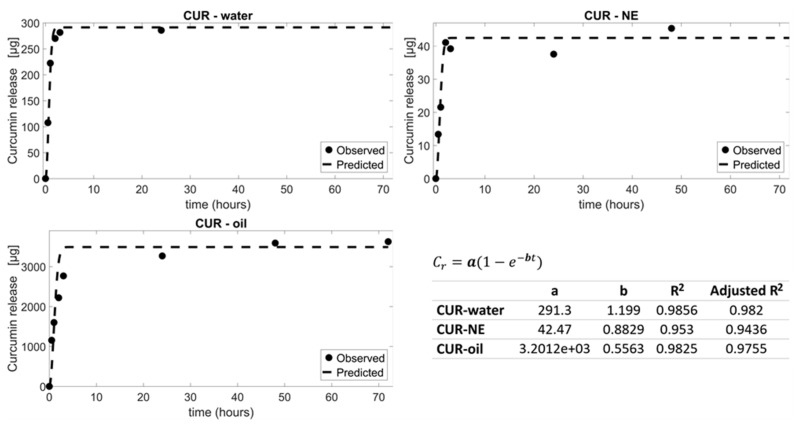
CUR–water, CUR–oil and CUR–NE experimental data were fitted with non-linear first-order models (dashed lines). The corresponding fitting parameters a and b are shownin the table as well as the R^2^ and adjusted R^2^ values.

For measuring curcumin release kinetics, the experimental release data of the three MP formulations were fitted using a non-linear first-order equation. Data fittings are shown in Figure 4 together with the extracted model parameters a and b. The correlation coefficient R^2^ and adjusted R^2^ values of CUR–water, CUR–NE and CUR–oil were 0.99, 0.95, 0.98 and 0.98, 0.94 and 0.98, respectively. Therefore, the experimental data were not far from the calculated ones, indicating the suitability of the non-linear first-order kinetic equation model. 

**Figure 5:**
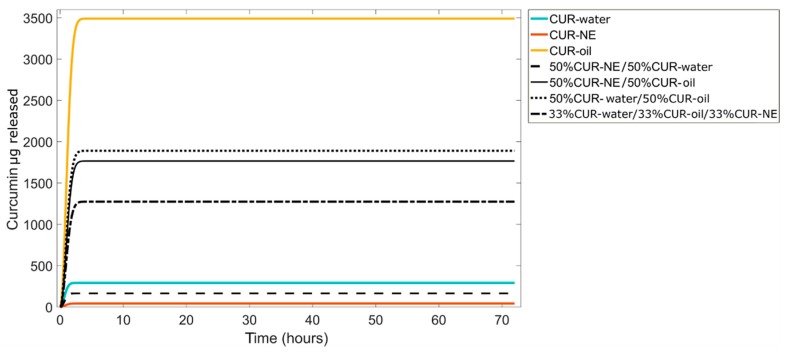
Predictive curcumin release kinetics can be obtained combining different CUR–water, CUR–oil and CUR–NE amounts by non-linear first-order models.

Once acquired the mathematical equations which describe the dependence of release as a function of time, a quantitative combination of non-linear first-order models (Equation (2)) describing the three CUR–MP kinetics was used for simulating further releases of the encapsulated molecule. Some possible combinations are shown in Figure 5. Mathematical modeling of curcumin release kinetics has been used to design a number of controlled MP-based drug delivery systems in order to release a specific concentration of curcumin in the target tissues with the desired timing. This tool is very useful to predict releases, avoiding the necessity of realizing experiments.

#### 3.3.2. In Vitro CUR Release

**Figure 6:**
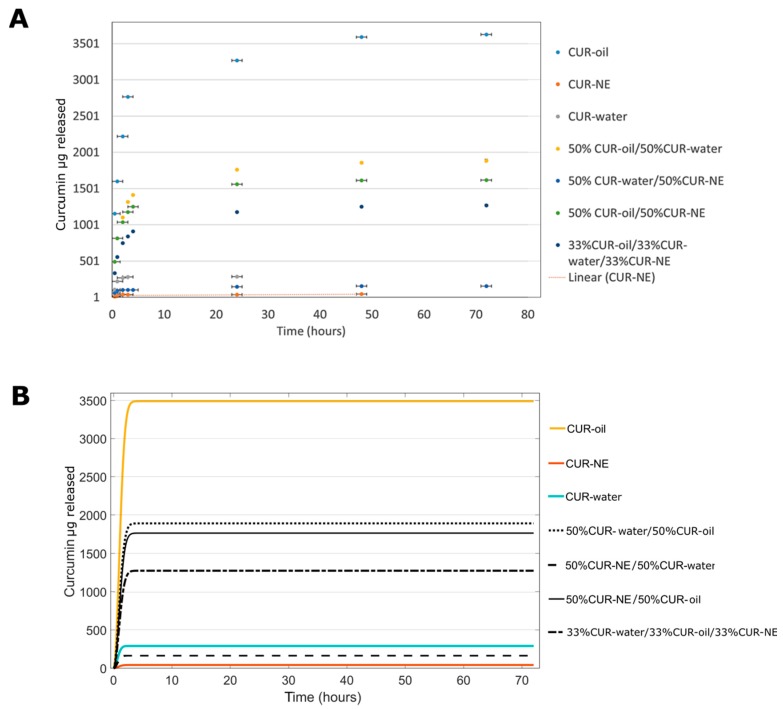
Correlation of curcumin released from different MP combinations: (**A**) experimental in vitro release, in PBS at pH 7.2, 550 rpm and 37 °C and (**B**) in silico release.

With the aim to understand the reliability and accuracy of the in silico studies, experimental in vitro release profiles of curcumin were performed. Particularly, four different combinations used for theoretical studies were analyzed: (i) 50% of CUR–oil plus 50% CUR–water, (ii) 50% of CUR–oil plus 50% CUR–NE, (iii) 50% of CUR–water–50% CUR–NE and (iv) 33% of all three formulations. Moreover, the release of curcumin from the single formulations was also evaluated as control. Interestingly, as shown in Figure 6A,B, a perfect correlation between hypothetical and experimental results was obtained, confirming that mathematical models can be a great support to reduce the number of experiments and to analyze different conditions and strategies. 

The amounts of curcumin released from each case coming from in silico and in vitro experiments after 72h, were summarized in Table 2. As we can see, the CUR–water and CUR–NE formulations can guarantee a fast release; after 72 h, all curcumin is released, but they are able to release only 285 ± 2.95 and 45.35 ± 4.21 μg of curcumin, respectively. In addition, for these formulations, a percentage of release more than 100% is reported; numbers over 100%, but still close to this value, are potentially due to random and systematic errors coming from the evaluation methods. Intermediary situations can be achieved by mixing them with CUR–oil configuration, indeed, using the 50% of CUR–oil MPs with 50% of CUR–water or CUR–NE, 60% of the drug can be released after 72 h with an amount of >1 mg. This situation was maintained even by using 33% of the three formulations together. The same results were confirmed by the in silico data (Table 2). The obtained outcomes underline how, thanks to our approach, we are able to finely regulate the quantity of the drug to be released, generating a powerful platform for the drug delivery field.

## 4. Conclusions

This project was undertaken to design curcumin-loaded PLGA MP-based formulations with tunable kinetic release by using a combination of different PLGA MPs. We demonstrated that the rate of curcumin released PLGA MPs can be controlled by playing on the encapsulation strategy which has an effect on the MP microstructure and on the drug distribution within the MPs. Moreover, thanks to the use of a non-linear first-order mathematical model we were able to predict and obtain intermediate situations capable of guaranteeing prolonged or fast drug releases by combining the starting PLGA MPs. The perfect agreement obtained between experimental and in silico methods, confirmed that mathematical modeling could be a valuable support to reduce the number of experiments during the development of novel personalized therapies, especially for the long-term ones. 

Our approach can be easily extended to other molecules besides curcumin and it will be useful to release drugs to the target sites with a controlled timing and amount, maximizing the therapeutic efficiency and thus decreasing the side effects.

## Figures and Tables

**Table 1:** %ɳ of CUR–MPs.

MPs	%ɳ ± SD
CUR–NE	31.02 ± 0.5
CUR–oil	40.01 ± 0.3
CUR–water	42.30 ± 3.5

**Table 2:** In silico and in vitro curcumin release experiments (*n* = 3).

MPs	µg of Curcumin Released In Silico (72 h)	% of Curcumin Released In Silico (72 h)	µg of Curcumin Released In Vitro (72 h)	% of Curcumin Released In Vitro (72 h)
CUR–oil	3491	77.4	3626±15	80±10
CUR–water	291.3	107.4	285±3	105±4
CUR–NE	42.47	99.2	45±4	106±3
50%CUR–oil/50%CUR–water	1891	64.7	1886±16	64±16
50%CUR–oil/50%CUR–NE	1766	63.3	1617±0.3	58±9
50%CUR–water/50%CUR–NE	166	95.6	155±2	89±12
33%CUR–oil/33%CUR water/33%CUR–NE	1275	62.4	1270±15	62±14
